# Current News

**Published:** 1898-02

**Authors:** 


### Current News.

### TENTH ANNIVERSARY OF THE ODONTOGRAPHIC SOCIETY OF CHICAGO.

The Odontographic Society of Chicago, the largest dental association in the United States excepting the American Dental Association, will celebrate its tenth anniversary February 21 and 22, 1898.

We shall spare no effort to make the event memorable in every respect, and to that end have planned a convocation which shall extend over two days, and consist of clinics and scientific papers from men specially distinguished and representative in the science and dentistry.

C. E. Bentley,
*Chairman Executive Committee.*

100 State Street, Chicago, Ill.

### ODONTOGRAPHIC SOCIETY OF CHICAGO.

The election of officers for the ensuing year of the Odontographic Society of Chicago resulted as follows:

President, G. W. Schwartz; Vice-President, H. J. Goslee; Secretary, F. H. Zinn, 70 State Street; Treasurer, George N. West.

*Member of Board of Directors*, B. J. Cigrand.

*Board of Censors.*—E. K. Bennington, A. G. Johnson, F. E. Roach.

